# Blood serum and BSA, but neither red blood cells nor hemoglobin can support vitellogenesis and egg production in the dengue vector *Aedes aegypti*

**DOI:** 10.7717/peerj.938

**Published:** 2015-05-05

**Authors:** Kristina K. Gonzales, Hitoshi Tsujimoto, Immo A. Hansen

**Affiliations:** 1 Department of Biology, New Mexico State University, Las Cruces, NM, USA; 2 Institute for Applied Biosciences, New Mexico State University, Las Cruces, NM, USA; 3 Molecular Biology Program, New Mexico State University, Las Cruces, NM, USA

**Keywords:** Artificial blood meal, *Aedes aegypti*, Vitellogenesis, Egg development, Mosquito culture, Mass rearing

## Abstract

*Aedes aegypti* is the major vector of dengue, yellow fever and chikungunya viruses that put millions of people in endemic countries at risk. Mass rearing of this mosquito is crucial for strategies that use modified insects to reduce vector populations and transmission of pathogens, such as sterile insect technique or population replacement. A major problem for vector mosquito mass rearing is the requirement of vertebrate blood for egg production since it poses significant costs as well as potential health hazards. Also, regulations for human and animal use as blood source can pose a significant obstacle. A completely artificial diet that supports egg production in vector mosquitoes can solve this problem. In this study, we compared different blood fractions, serum and red blood cells, as dietary protein sources for mosquito egg production. We also tested artificial diets made from commercially available blood proteins (bovine serum albumin (BSA) and hemoglobin). We found that *Ae. aegypti* performed vitellogenesis and produced eggs when given whole bovine blood, serum, or an artificial diet containing BSA. Conversely, egg production was impaired after feeding of the red blood cell fraction or an artificial diet containing only hemoglobin. We also found that egg viability of serum-fed mosquitoes were comparable to that of whole blood and an iron supplemented BSA meal produced more viable eggs than a meal containing BSA alone. Our results indicate that serum proteins, not hemoglobin, may replace vertebrate blood in artificial diets for mass mosquito rearing.

## Introduction

During the past decades, several mosquito control strategies have been developed that require the release of large numbers of mosquitoes grown in culture: 1. Sterile Insect Technique (SIT) requires the production of large quantities of male mosquitoes that are sterilized either by ionizing radiation (Helinski, Parker & Knols, 2009; Offori & Van der Vloedt, 1990; Rodriguez et al., 2013) or that are genetically sterile (Alphey, 2002; Alphey, 2007); 2. Infection with endosymbiotic bacteria, *Wolbachia* can make mosquitoes refractory to viral infections (Moreira et al., 2009; Walker et al., 2011). In order to drive these endosymbionts in field populations, large numbers of *Wolbachia*-infected females are needed (Rainey et al., 2014). 3. For replacement of field populations with modified mosquitoes that are refractory for pathogens, both males and females can be released.

Mosquitoes are hematophagous insects, and only certain species require a blood meal to produce their first batch of eggs. Such mosquitoes are termed anautogenous. In contrast autogenous mosquitoes can produce a first batch of eggs using nutrients acquired from their larval stage and require blood only to produce subsequent egg batches (Hansen et al., 2014). Since anautogenous mosquitoes need vertebrate blood for egg development, and most vector species are anautogenous, a source of blood and an efficient feeding system has to be acquired at every facility mass-rearing mosquitoes for the above mentioned strategies. This can pose a significant hindrance since local regulations, ethical concerns, and infrastructure vary greatly in different countries. In many countries, protocols involving animal subjects have to undergo a review process leading to an animal care and use program before experiments are carried out (Anderson et al., 2002). This can result in significant delays in laboratory activity and added requirements, recordkeeping and personnel training. Therefore, the push for alternative vertebrate blood-free meals is attractive to mosquito rearing facilities all over the world. In order to replace vertebrate blood in mosquito rearing, an artificial blood meal has to meet the following requirements: (1) Mosquito females must readily take it in sufficient amounts, (2) it must support vitellogenesis, (3) it must support large egg batches, and (4) the offspring should be fit.

Vertebrate blood is a mixture of erythrocytes, leucocytes and platelets suspended in an aqueous medium called plasma. Erythrocytes (red blood cells: RBCs) are the main component of blood comprising ∼45% of total blood volume and the major protein in these cells is hemoglobin (Hb). The other 55% of blood consists of plasma, which is a water- and protein-rich formulation that has a balanced salt concentration acting as a buffer to maintain stable pH levels and other cellular components. Nutrients absorbed into the blood stream from digested food, dissolved gases and other blood proteins and lipids are also found in blood plasma (Farley, Hendry & McLafferty, 2012). Blood can be fractionated into packed red blood cells and plasma, using centrifugation protocols (Duarte, Carvalho Simões & Sgarbieri, 1999).

*Ae. aegypti* is the primary vector for Yellow fever, Dengue and Chikungunya viruses through saliva during blood feeding (Go, Balasuriya & Lee, 2014). *Ae. aegypti* is an invasive species in the Americas, preferring areas close to humans where blood is easily accessible (Powell & Tabachnick, 2013). After a female takes a blood meal into her midgut, the blood proteins are enzymatically digested into amino acids, which are then released into the hemolymph (Pacey & O’Donnell, 2014). The accumulated amino acids in the hemolymph are absorbed by the mosquito fat body, functionally similar to the vertebrate liver, which synthesizes yolk protein precursors (YPP), called vitellogenin. These YPPs are then secreted into the hemolymph and taken up by developing oocytes via a vitellogenin receptor (Sappington, Hays & Raikhel, 1995), a process called vitellogenesis (Hansen et al., 2014).

A study on blood substitutes for *Ae. aegypti* found that feeding a mixture of blood proteins can support egg production in *Ae. aegypti*. Increasing the protein content from 60 mg/ml to 123 mg/ml of a mixture of porcine albumin, Hb, and *γ*-globulins in the artificial meal significantly increased the number of deposited eggs (Kogan, 1990). Another recent study presents an artificial diet based on two concentrations, 100 and 200 mg/ml, of bovine serum albumin (BSA) that can support *Ae. albopictus* vitellogenesis and egg development (Pitts, 2014). The total number of eggs produced from the 100 mg/ml BSA meal was statistically significantly reduced compared to the number of eggs produced from whole blood and a 200 mg/ml BSA meal. The findings of these studies provide important considerations for the development of artificial blood meals for mosquitoes.

In addition, phagostimulants are important components of artificial blood meals because they increase the proportion of females that take the meal and the amount of meal taken. Studies focused on identifying phagostimulants found that mosquitoes were likely to fully engorge on meals containing adenyl nucleotides (adenosine monophosphate (AMP), adenosine diphosphate (ADP), and adenosine triphosphate (ATP)) and the success rate increased with the number of attached phosphate groups (Clements, 1992). A phagostimulant study produced similar results when bed bugs, *Cimex lectularius*, were offered 1 mM of AMP, ADP and ATP (Romero & Schal, 2014). 1 mM of ATP has also been shown to be the most effective concentration in *Ae. albopictus* feeding experiments (Pitts, 2014).

The aim of the present study was to develop a blood-free meal that supports egg development in *Ae. aegypti*. We have tested different protein components, BSA, Hb, serum and red blood cells for their effect on egg production. We found that neither RBC nor Hb, but blood serum proteins are sufficient to support egg production in *Ae. aegypti*.

## Materials and Methods

**Insect culture.** *Ae. aegypti* (Rockefeller strain) eggs were submerged in tap water and connected to a vacuum pump for 20 min to deoxygenate the water and induce hatching. Larvae were raised on cat food (Special Kitty; Walmart, Bentonville, Arkansas, USA) and water was changed as needed. Pupae were separated by hand using a handheld screen and placed in a separate container with clean water. Pupae containers were placed inside 30 × 30 × 30 cm cube-shaped mosquito cages for emergence with 20% sucrose solution as carbohydrate source. During the rearing process mosquitoes were held in an insect environmental chamber at 26.5 °C, 70% relative humidity (RH) and 16:8 h light/dark cycle. Adults were at least 4 days old before given a meal.

**Meal treatments.** Prior to feeding experiments, sucrose solution was withheld from mosquitoes for at least 16 h. Starved females were separated from males and equally distributed into smaller 15 × 15 × 15 cm cube-shaped mosquito cages and fed using an artificial lab-made feeding system. RBCs or blood serum was fractionated from whole defibrinated bovine blood (Hemostat Labs, Dixon, California, USA). Whole blood and serum was given alone and RBCs were washed with phosphate buffered saline (PBS). The whole blood was centrifuged and the serum supernatant was pipetted off without disturbing packed RBCs. Serum was collected and stored at −20 °C. RBCs were washed 3 times by resuspending in one volume of PBS, centrifuging and pipetting off supernatant. Washed RBCs were stored at 4 °C. All centrifugations were done at ∼1,275 × *g* for 20 min at 4 °C. Females were each fed a meal consisting of different protein components. BSA (Research Products International, Mt. Prospect, Illinois, USA) or Hb (Sigma-Aldrich, St. Louis, Missouri, USA) (200 mg/mL) meal was dissolved in either PBS, *Aedes* physiological saline (APS), sodium PBS (NaPBS) or potassium PBS (KPBS). Chemical concentrations and components are listed in Table 1. A 50 mM ATP (Sigma-Aldrich, Seelze, Germany) solution was made and immediately stored at −20 °C. ATP solution was thawed right before feeding experiment and kept on ice during preparation.

**Table 1:** Chemical components and pH of each buffer used in BSA and Hb feedings. Phosphate buffered saline (PBS), *Aedes* physiological saline (APS), Sodium phosphate buffered saline (NaPBS), and potassium phosphate buffered saline (KPBS). All concentrations are in mM.

	PBS	APS	NaPBS	KPBS
Sodium Chloride (NaCl)	136.9	150.0	137.0	2.7
Potassium Chloride (KCl)	2.7	4.0	2.7	137.0
Disodium Phosphate (Na_2_HPO_4_)	8.1	0.0	10.0	2.0
Sodium Bicarbonate (NaHCO_3_)	0.0	0.1	0.0	0.0
Magnesium Chloride (MgCl_2_)	0.0	0.6	0.0	0.0
Potassium Dihydrogen Phosphate (KH_2_PO_4_)	1.5	0.0	2.0	10.0
Calcium Chloride (CaCl_2_)	0.0	1.7	0.0	0.0
HEPES buffer	0.0	25.0	0.0	0.0
pH	7.4	7.0	7.5	7.5

**Figure 1:**
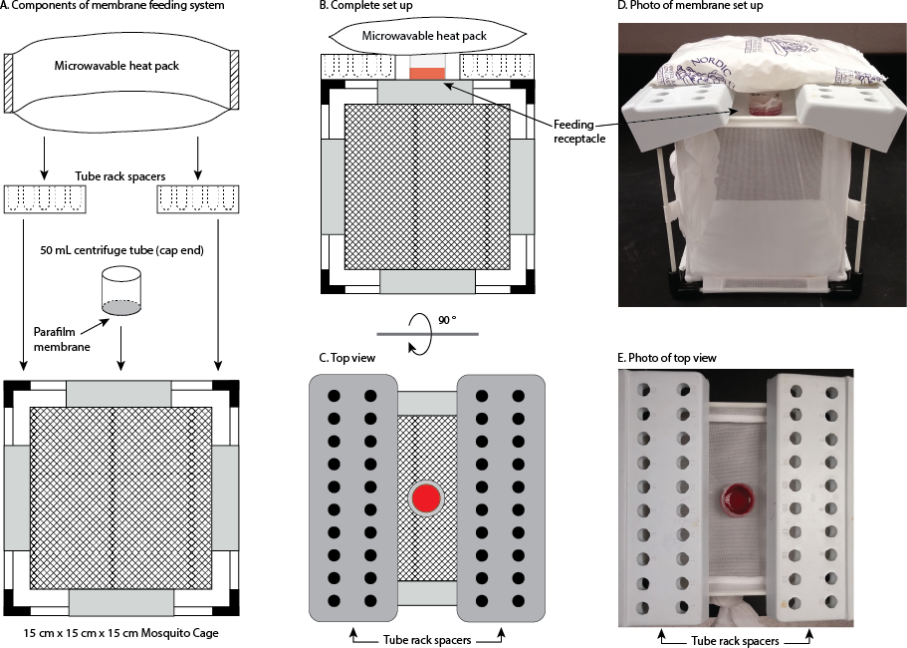
Membrane feeding system. (A) Components of the membrane feeding system which are: microwavable heat packs, tube rack spacers, the feeding receptacle with parafilm membrane placed on top of a 15 × 15 × 15 cm cage. (B) Complete set up of all components of the feeding system. (C) Top view of the feeding system (rotated 90°) showing the tube rack spacers with feeding receptacle filled with whole defibrinated bovine blood (red center). (D) Photograph of the complete set up of the membrane feeding system seen from the side. (E) Photograph of membrane system as seen from the top with tube rack spacers and feeding receptacle filled with whole defibrinated bovine blood.

**Membrane feeding system.** A membrane feeding system was constructed from available laboratory materials (Fig. 1). A cylindrical feeding receptacle was made from a 50 mL centrifuge tube that was cut at the 45 mL marking. One open end of the receptacle was sealed with parafilm stretched to near breaking point to serve as a membrane for mosquito piercing mouthparts. The parafilm membrane was rubbed on sweaty human skin to stimulate host-seeking behavior. 1 mL of each prepared meal solution was heated in a 37 °C water bath for 15 min, then pipetted into the feeding receptacle and placed, parafilm side down, on top of each 15 × 15 × 15 cm cage. ATP was added to a final concentration of 1 mM as a phagostimulant in all treatment meals. Re-usable heat packs were microwaved for 2 min and placed on top of each feeding receptacle to keep solution warm and to aid in attracting mosquitoes. A microcentrifuge tube rack was placed on each side of the feeding receptacle to provide support for the packs. Females were provided with a meal for 1 h then immediately collected with a battery operated aspirator and anesthetized on ice. Fully engorged females were counted and weighed together on a balance. The females were then relocated to a different 15 cm × 15 cm × 15 cm cube-shaped mosquito cage, provided with a water-soaked cotton ball and a 20% sucrose-soaked cotton ball on top, and kept at 26.5 °C, 69% RH for 48 h. Each female was then individually placed into a 50 mL centrifuge tube containing a water-soaked cotton ball and filter paper substrate for egg deposition for additional 24 h. Seventy-two hour post-fed dissections were carried out and the number of fully-developed retained oocytes and the number of deposited eggs were determined.

**Egg viability test.** To determine egg viability of the deposited eggs, an *en masse* viability test was performed. Groups of 20 individual females were starved for at least 16 h and fed whole blood, serum, 200 mg/ml BSA in APS, and an iron-enriched BSA in APS solution (200 mg/ml BSA in APS + 0.5 mg/mL Iron (III) chloride). The iron concentration was selected because it reflects the average iron content present in whole blood of normal individuals (Helmer & Emerson, 1934). The females were allowed to lay eggs at 72 h post blood meal for 24 h. Ninety-six hours post blood meal, the eggs of each group were removed from the cage and kept on a moist filter paper for the next 48 h to allow them to mature. After 48 h the eggs were counted and stored in paper envelopes for another 24 h in an insect environmental chamber. Three days after deposition, the eggs were placed in 100 mL of deionized water and connected to a vacuum pump for 30 min to induce hatching. Vacuumed eggs were transferred to shallow plastic trays and provided with Special Kitty food pellets and placed inside an insect environment chamber. Egg viability was determined by counting the number of larvae present 5 days after the eggs being placed in water.

**Data analysis.** A Kruskal–Wallis analysis of variance was conducted between feeding trials of the same meal to detect any significant variation. If no significant variation was discovered, then the trials were grouped and compared to other protein meals. The proportion of females that fed was calculated by dividing the number of fully engorged females by the total number of females given the meal. The Kruskal–Wallis test was performed to determine significant differences among multiple groups, and the Mann–Whitney U test was used to compare pairwise treatment meals. In the analysis of egg deposition numbers of all treatment meals, the high percentage of zero values for the RBC and Hb treatments, and consequent high number of ties, precluded the use of the U test for pairwise comparisons. Therefore, data for whole blood control and all other treatments were recoded as “yes” for individual females that laid eggs and “no” for individual females that did not. A contingency table analysis, Fisher’s exact test, was used to determine significant differences between each meal. For egg viability, the proportion of viable eggs was calculated by dividing the number of larvae present after 5 days (indicates the hatch rate) by the total number of eggs deposited for each treatment meal. A total of four replicates was conducted. Each decimal fraction was transformed into arc sine data (arc sine of the square root of each decimal fraction) and analyzed by a one-way analysis of variance (ANOVA) test to detect statistical significant differences between treatment meals with the Holm–Sidak posttest for pairwise comparisons versus the whole blood control. Some statistical tests were performed on JMP 12.0 (SAS Institute, Inc., Cary, North Carolina, USA) software and others on SigmaPlot 12.0 (Systat Software, Inc., San Jose, California, USA). Graphs were made using SigmaPlot 12.0 and Adobe Illustrator Creative Cloud (Adobe Systems, Inc., San Jose, California, USA).

## Results & Discussion

The current study was conducted in order to identify the necessary nutritional requirements for mosquito egg production to ultimately replace traditional vertebrate blood feeding protocols in mosquito rearing facilities. An effective artificial blood meal has to meet the four requirements described in the “Introduction.”

**Figure 2:**
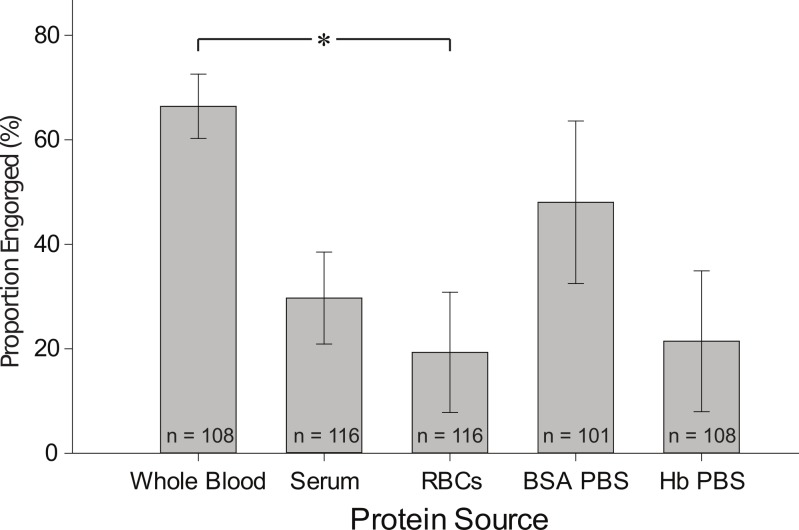
Proportion of fully engorged females fed on whole blood, serum, RBCs, BSA in PBS and Hb in PBS. Data is representative of at least three independent trials. Graph represents mean ± SEM; *n* = number of females given the meal. The Mann-Whitney test was performed to determine statistical differences in comparison to whole blood: ∗ indicates significant difference at *P* < 0.05.

*Mosquitoes prefer buffered BSA solution and serum over red blood cells.* To address whether an alternative artificial protein formulation could be taken in sufficient amounts and support vitellogenesis, we offered several diet formulations containing different protein sources to *Ae. aegypti* females: BSA, and Hb constituted in PBS, RBCs, serum, and whole blood as control. We determined the proportion of fully engorged females after one hour (Fig. 2). The highest proportion of females fully engorged was found with whole blood (control) and this value was significantly different from the number of females engorged on RBCs. The percentage of mosquitoes engorged on serum, BSA, and Hb solutions was not quite significantly different from the control. The engorgement rates were variable in some of the trials conducted which may have been the result of utilizing different cohorts of females and feeding on different days and times of the day. However, a strong trend was found that the BSA solution is the preferred meal, when compared to the other protein sources. The RBC and Hb formulation had the lowest engorgement response which consequently resulted in a small sample size when statistical analysis was performed and compared to the other protein sources. However, independent from the diet formulations offered, mosquitoes were in all cases actively probing the membranes of each feeding apparatus. It is apparent that mosquito females, when they come in physical contact with the diet solution, make a decision either to continue feeding or reject the meal and continue probing. One possible explanation for the reduced engorgement rates we observed with some diet formulations is that RBCs and Hb somehow made the phagostimulant ATP inaccessible for the mosquito either by binding it or by chemical conversion. Another, and in our opinion more likely explanation is that mosquitos can taste the solution with chemoreceptors that are located in the labella or, most likely in the cibarium (Clements, 1992). In general, our results support the idea that mosquitoes have a system to judge the quality of a blood (food) source, and based on this information, accept or reject it. The nature of the sensing system is unknown and an interesting topic for further studies.

**Figure 3:**
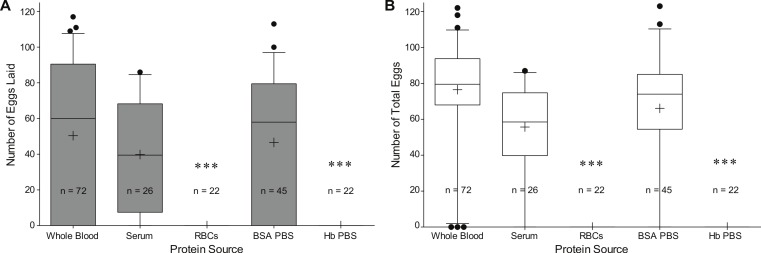
*Ae. aegypti* egg production after feeding on different diets. (A) Number of eggs laid; (B) total number of eggs (laid and fully developed in females but not laid (retained)). Horizontal bars represent the median, and “+” as mean with 25th and 75th percentiles represented by boxes. Whiskers extend to the 5% and 95% range and individual circles are outliers. *n* = number of females engorged with the meal. Each treatment meal was analyzed with each other using a contingency table analysis (Fisher’s exact test). ∗∗∗ denotes statistical significant difference (*P* < 0.0001) in comparison to other sample groups without the same notation.

**Figure 4:**
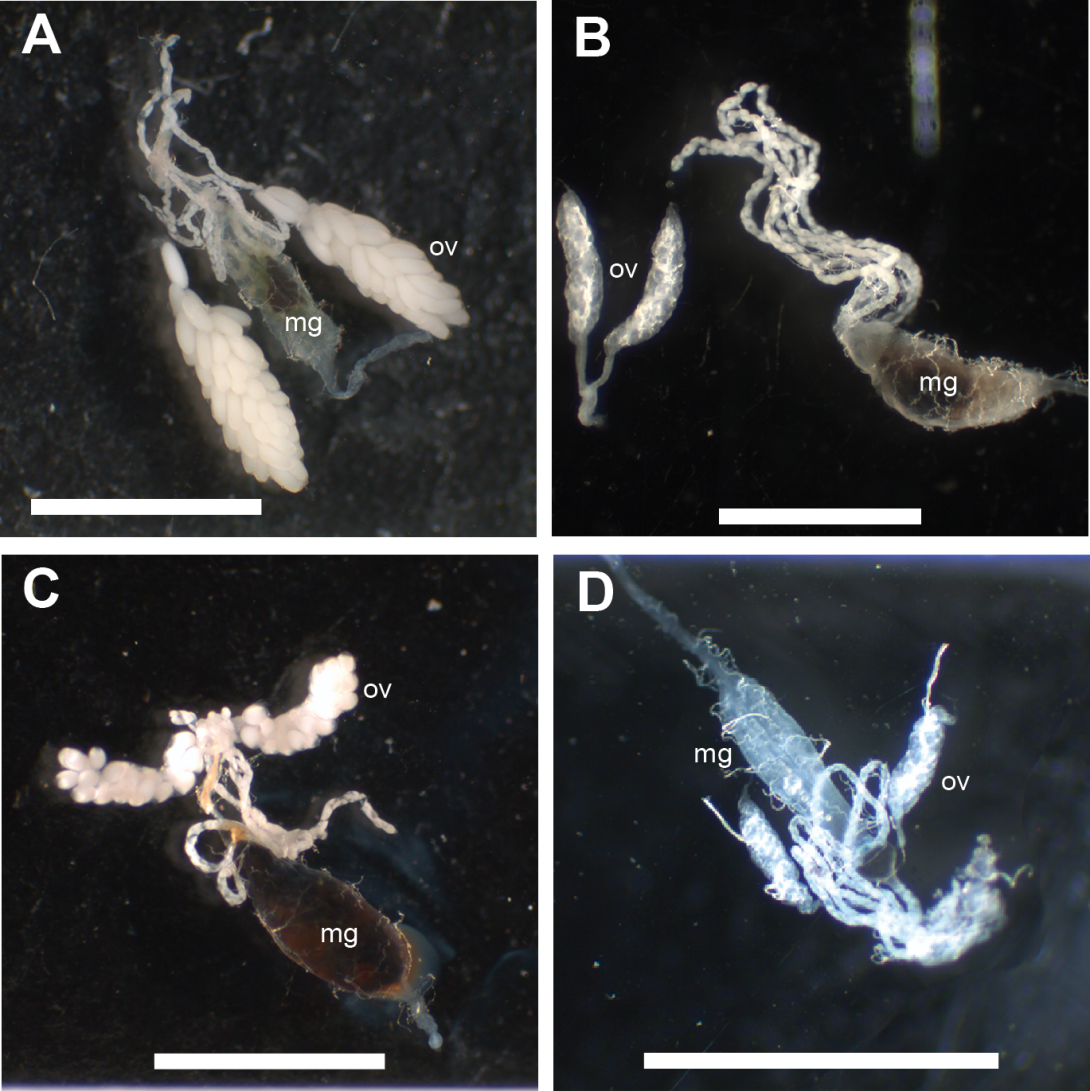
The effect of whole blood, RBCs and Hb meals on ovary development 72 h after feeding. Midguts with attached Malpighian tubules and ovaries were dissected: mg, midgut; ov, ovary. Scale bars = 2 mm. (A) Fully developed ovaries from a female given whole blood (control), (B) No ovary development in females fed with PBS-buffered Hb diet, (C) Partial ovary development seen in a few females given RBC as sole protein source, (D) Unfed control female.

*Red blood cells or hemoglobin do not support egg production.* To determine if our artificial blood meal can produce large egg batches, we tested the effects of feeding different diets on egg deposition rates. We compared whole blood (positive control), against its fractionated components, serum and RBCs, and both BSA and Hb in PBS (Fig. 3). An initial Kruskal–Wallis analysis of variance test revealed a significant difference in egg production among the five treatments (*DF* = 4, *H* = 61.735, *P* < 0.0001 for laid eggs; *DF* = 4, *H* = 97.310, *P* < 0.0001 for total eggs). Each treatment was compared with each other using a contingency table analysis (Fisher’s exact test). We found a statistical significant difference between RBC or Hb and other sample groups (*P* < 0.0001), but neither between RBC and Hb, nor between any combination of other groups. Notably, no egg was laid by females fed on the RBC or Hb meal. Most of the oocytes in whole-blood fed females developed fully (Fig. 4A), while most of those in RBC or Hb-fed females did not (Figs. 4B and 4C). Partial oocyte development was observed in two out of 22 females that fed on the RBC formulation (Fig. 4C) and in zero out of 22 females that fed on the Hb formulation (Fig. 4B). We also observed what appears to be a mostly undigested bolus of RBCs and Hb in the midgut of the RBC-fed and Hb-fed females (Figs. 4B and 4C). Our results indicate that a diet of pure RBCs and Hb causes a delayed or early aborted digestion compared to whole blood-, serum-, or BSA-fed females. This phenomenon may be due to elevated load of iron associated with RBC or Hb, although under natural conditions some of the iron from a blood meal is packaged in eggs, while most of it is excreted. Interestingly, a blood meal seems to be detrimental to mosquitoes in the absence of an antioxidant, xanthurenic acid (Lima et al., 2012). Some species of mosquitoes have one or two rows of teeth of cuticle called the cibarial armature within their cibarium (preoral food cavity). This structure and surrounding structures function to lyse RBCs. Interestingly, *Ae. aegypti* lacks the cibarial armature and lyse only 3%-5% of RBCs. Its saliva and gut extract do not lyse RBCs, which implies that *Ae. aegypti* selectively minimizes digestion of RBCs (Coluzzi, Concetti & Ascoli, 1982). Yet another possibility is that the different meals modified the mosquito’s gut microbiota in different ways as shown in an earlier study (Oliveira et al., 2011). In the case of RBC and Hb this change could be unfavorable for egg development.

**Figure 5:**
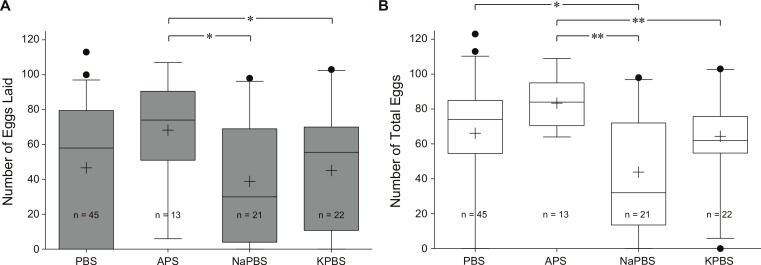
The effect of different buffer solutions on egg deposition provided a 200 mg/ml BSA meal. Box plots showing the number of eggs laid (A) and total eggs (B) per female between 48 and 72 h post-meal. Each column represents BSA delivered in PBS, APS, NaPBS, and KPBS. *n* = number of females engorged with the meal. Horizontal bars represent the medians with 25th and 75th percentiles represented by boxes. “+” represents mean. Whiskers extend to the 5% and 95% range and individual circles are outliers. ∗ and ∗∗ denote statistical significance at *P* < 0.05 and *P* < 0.01 by Mann-Whitney U test, respectively.

*Effect of different buffers on egg production after a BSA meal.* To further analyze whether our protein meals could produce large egg batches, we offered a BSA meal dissolved in four different buffers. After a blood meal, *Ae. aegypti* mosquitoes excrete up to 80% of the meal volume within the first hour (Drake et al., 2010; Drake et al., 2012). The meal becomes more concentrated and therefore easier to digest and the mosquito loses weight which restores its flight capabilities. We hypothesized that an efficient concentration process after an artificial BSA meal depends on the ion concentrations of the buffer and can be optimized. In order to determine a good buffer solution for a blood-free meal we offered BSA meals in four different buffer solutions: Phosphate-buffered saline (PBS), *Aedes* physiological saline (APS), Sodium PBS (NaPBS) and Potassium PBS (KPBS). We used 200 mg/ml BSA in the various buffers since this concentration supported *Ae. aegypti* vitellogenesis in the experiments described above and also has been shown to support egg development in *Ae. albopictus* (Pitts, 2014). We found a statistical significant difference between APS and NaPBS or KPBS in egg deposition (Fig. 5). We also found a statistical significant, yet lower degree, difference between PBS and NaPBS in total egg number. Although the chemical composition of the PBS and NaPBS are similar, their effects on the number of total eggs produced (laid and retained) were statistically significantly different. There was no difference in observed mosquito probing activity between the two solutions; however, the amount of BSA PBS solution was taken in larger amounts per female, and with overall greater engorgement rates, than the BSA NaPBS solution in all feeding replicates (average 3.7 mg/female, 48.04%; average 2.8 mg/female, 25.0% respectively). There is no clear explanation for this occurrence. A slight increase in millimolar concentration of disodium phosphate in the NaPBS buffer could account for differences seen in total egg numbers between BSA PBS and BSA NaPBS. However, we did not find difference between PBS and APS. The results suggest that change in cationic components (Na^+^ and K^+^) affects mosquito’s egg production. We also offered Hb meals in the four different buffer solutions to compare with BSA meals. Hb did not support egg production in any buffer solution (Table 2).

**Table 2:** *Aedes aegypti* egg production response to BSA and hemoglobin meal in four different buffers. Mean numbers of eggs laid and retained in the ovaries ±SEM per female are shown. Representative of three independent replicated feedings. *n* = number of engorged females for each meal.

		PBS	APS	NaPBS	KPBS
Bovine Serum Albumin	*n*	26	9	18	6
	Eggs laid	42 ± 8.01	66 ± 10.80	37 ± 7.98	52 ± 17.02
	Eggs in ovaries	23 ± 7.96	21 ± 10.29	6 ± 3.85	25 ± 11.34
Hemoglobin	*n*	18	7	17	17
	Eggs laid	0 ± 0.00	0 ± 0.00	0 ± 0.00	0 ± 0.00
	Eggs in ovaries	0 ± 0.00	0 ± 0.00	1 ± 1.00	0 ± 0.00

**Figure 6:**
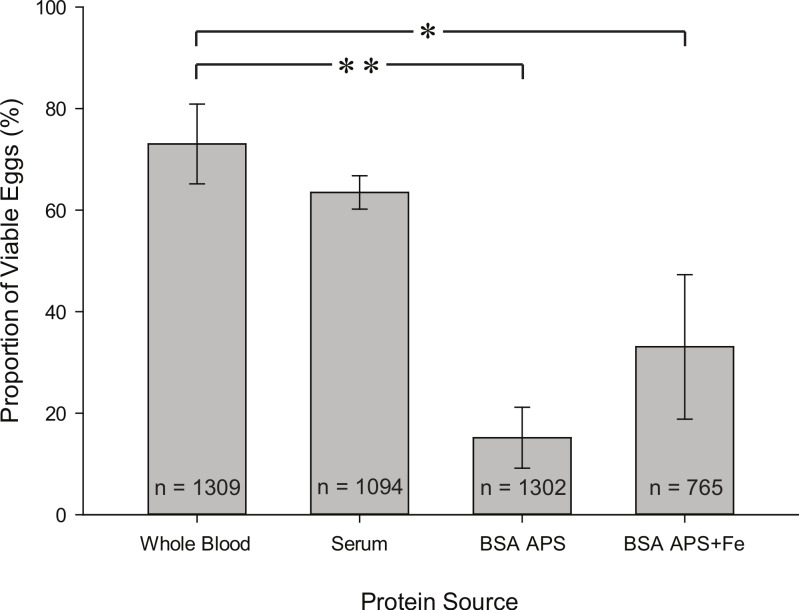
Proportion of viable eggs from females fed on whole blood, serum, BSA APS, and BSA APS + Fe. Graph represents mean ± SEM of four trials. A One-way ANOVA test was performed to determine statistical differences between treatment meals versus the whole blood control (Holm–Sidak posttest): ∗ and ∗∗ denote statistical significance at *P* < 0.05 and *P* < 0.01, respectively. *n* = the number of eggs laid per treatment.

*Iron supplementation plays a role in egg viability.* To assess offspring numbers produced with different diets, we performed an egg viability test on whole blood (control), serum, BSA in APS and an iron-enriched version of BSA APS (Fig. 6). An iron (III) chloride-enriched (0.5 mg/ml) BSA APS solution was added in order to determine whether iron supplementation can enhance egg viability. We found that the proportion of viable eggs in the serum treatment meal is not statistically significantly different from whole blood. We also found that there are statistical significant differences between whole blood and the BSA APS (*DF* = 3, *P* = 0.002, *T* = 4.663) and BSA APS + Fe (*DF* = 3, *P* = 0.018, *T* = 3.115) treatment meals. We hypothesized that the BSA APS solution would be the best candidate for an alternative blood meal. BSA APS met the first three requirements of a successful artificial blood meal (see ‘Introduction’); however, the reduced hatching rates imply that there might be a problem with the last requirement of offspring fitness (4. The offspring should be fit). The proportion of viable eggs was roughly 1/5 of that from the whole blood control which suggests that BSA APS meal is missing some components for efficient embryonic development pre-eclosion. Interestingly, feeding the BSA APS + Fe meal resulted in an increased proportion (∼2x) of viable eggs when compared to the results obtained by feeding BSA APS meal (Fig. 6). The iron concentration we chose is representative of the average iron content found in normal human host individuals (Helmer & Emerson, 1934). However, the BSA APS + Fe meal was statistically significantly lower than that of whole blood. The positive effect of iron supplementation implies that iron plays a role in efficient embryonic development. Nevertheless, our results on egg production strongly suggest that despite high iron concentrations, RBC or Hb are not suitable protein sources for mosquito vitellogenesis and egg production. This could be explained by the fact that heme-associated iron cannot be utilized by *Ae. aegypti* for egg production, but iron in other forms (e.g., iron with transferrin found in serum) can be utilized (Zhou et al., 2007).

Our observations suggest that iron supplementation had a positive effect on egg viability. Further experiments with different iron preparations and other micronutrients are necessary to optimize artificial blood meal formulations.

## Conclusion

The goal of this study was to identify potential alternatives to vertebrate blood meals and test the hypothesis that such meals can support egg production in the dengue vector, *Ae. aegypti*. We were able to show that vitellogenesis and egg production can be supported on serum fractions from whole blood and PBS-buffered or APS-buffered BSA solutions. The egg numbers produced by females fed on such diets are comparable to whole blood. We also found that balance in cations in meal buffer affects egg production of the mosquito and that egg viability may have a positive correlation with iron and other micronutrients. Our findings are important for further development of blood meal alternatives in order to replace traditional vertebrate feeding protocols in mosquito mass culture.

## Supplemental Information

10.7717/peerj.938/supp-1 Supplemental Information 1 Raw data
